# Letter to the editor concerning “Current state of preoperative embolization for spinal metastasis – A survey by the EANS spine section” by Stefan Motov et al.

**DOI:** 10.1016/j.bas.2024.102788

**Published:** 2024-03-20

**Authors:** Tomohito Yoshihara, Tadatsugu Morimoto, Hirohito Hirata, Masatsugu Tsukamoto, Masaaki Mawatari

**Affiliations:** Department of Orthopaedic Surgery, Faculty of Medicine, Saga University, 5-1-1 Nabeshima, Saga, Japan

We were intrigued by the article by Stefan Motov et al. (2023), which explored the significant role of preoperative embolization (PE) for spinal metastases and investigated the varied approaches to embolization employed by spine surgeons and institutions. This innovative research, the first of its kind, holds great value for spine surgeons confronting metastatic spinal tumors. However, we have some concerns regarding the study's methodology.

Our first concern relies on the questionnaire's low response rate of 4%. Despite the author's acknowledging this limitation, the significant number of missing responses in other questions (e.g., 43% missing data on PE timing) raises questions about the survey's overall effectiveness and the consequent sample quality. While the average response rate for online surveys can be as high as 44%, as cited in the paper (Wu et al., 2022), it's essential to consider the target population's sophistication and the potential impact of reminder features. In this case, targeting board-certified specialists exclusively might have yielded more consistent and actionable data.

Second, while the survey included board-certified specialists and residents affiliated with the European Association of Neurosurgical Societies, the emphasis on perceived effects of embolization and experience suggests that targeting only board-certified specialists would have yielded more valuable data. Including residents and medical students may have introduced unnecessary variability and bias into the results.

Third, given the critical importance of performing PE within 48 hours of surgery for spinal metastases, potential biases in the interventionist's workflow or the medical facility itself could significantly influence the study's outcomes.

Finally, the potential risk of spinal cord ischemia due Adamkiewicz artery embolization during PE at the thoracolumbar level has been documented (Becker et al., 2023). Could the specific vertebral level of the lesion (metastatic disease near the thoracolumbar junction) preclude PE in some cases? If so, did this study account for this potential limitation?

To enhance our understanding of your valuable research, we welcome your response to our feedback. We remain available to address any questions or comments you may have.

## IRB approval

None (because letter to the editor is not a research that must be approved by the IRB)

## Declaration of competing interest

The authors declare that they have no known competing financial interests or personal relationships that could have appeared to influence the work reported in this paper.
